# The attenuation of renal fibrosis by histone deacetylase inhibitors is associated with the plasticity of FOXP3^+^IL-17^+^ T cells

**DOI:** 10.1186/s12882-017-0630-6

**Published:** 2017-07-10

**Authors:** Wen-Pyng Wu, Yi-Giien Tsai, Tze-Yi Lin, Ming-Ju Wu, Ching-Yuang Lin

**Affiliations:** 1 Graduate Institute of Clinical Medical Science, College of Medicine, China Medical University, Taichung, Taiwan; 2 Division of Nephrology, Ching Chyuan Hospital, Taichung, Taiwan; 3 Department of Pediatrics, Changhua Christian Hospital, Changhua, Taiwan; 4 School of Medicine, Kaohsiung Medical University, Kaohsiung, Taiwan; 5 Department of pathology, China Medical University Hospital, Taichung, Taiwan; 6 School of Medicine, Chung-Shan Medical University, Taichung, Taiwan; 7 Division of Nephrology, Department of Medicine, Taichung Veterans General Hospital, No. 1650, Taiwan Boulevard Sect. 4, Taichung, 40705 Taiwan, Republic of China; 8 Institute of Clinical Medicine, National Yang Ming University, Taipei, Taiwan; 9 Graduate Institute of Biomedical Science, National Chung Hsing University, Taichung, Taiwan; 10 Clinical Immunological Center, China Medical University Hospital, No. 2, Yude Road, Taichung, 40447 Taiwan, Republic of China

**Keywords:** Unilateral ureteric obstruction, Renal fibrosis, Tgf-β, FOXP3^+^ IL-17^+^ T cells

## Abstract

**Background:**

The histone deacetylase (HDAC) inhibitor, which has potential effects on epigenetic modifications, had been reported to attenuate renal fibrosis. CD4^+^ forkhead box P3 (FOXP3)^+^ T regulatory (Treg) cells may be converted to inflammation-associated T helper 17 cells (Th17) with tissue fibrosis properties. The association between FOXP3^+^IL-17^+^ T cells and the attenuation of renal fibrosis by the HDAC inhibitor is not clear.

**Methods:**

This study evaluated the roles of the HDAC inhibitor, Treg cells and their differentiation into Th17 cells, which aggravate chronic inflammation and renal fibrosis in a unilateral ureteral obstruction (UUO) mouse model. The study groups included control and UUO mice that were monitored for 7, 14 or 21 days.

**Results:**

Juxtaglomerular (JG) hyperplasia, angiotensin II type 1 receptor (AT1R) expression and lymphocyte infiltration were observed in renal tissues after UUO but were decreased after trichostatin A (TSA) treatment, a HDAC inhibitor. The number of CD4^+^FOXP3^+^ T cells increased progressively, along with the number of FOXP3^+^interleukin (IL)-17^+^ T cells, after 14 days, and their numbers then progressively decreased with increasing CD4^+^IL-17^+^ T cell numbers, as demonstrated by double immunohistochemistry. Progressive renal fibrosis was associated with the loss of CD4^+^FOXP3^+^IL-17^+^ T cells in splenic single-cell suspensions. FOXP3^+^IL-17^+^ T cells expressed TGF-β1 both in vitro and in vivo*,* and TGF-β1 expression was significantly knockdown by IL-17 siRNA in vitro. These cells were found to play a role in converting Tregs into IL-17- and TGF-β1-producing cells.

**Conclusions:**

TSA treatment decreased JG hyperplasia, the percentage of FOXP3^+^IL-17^+^ cells and the degree of fibrosis, suggesting that therapeutic benefits may result from epigenetic modifications.

## Background

Renal fibrosis arises in most progressive renal diseases, regardless of the disease underlying end-stage kidney disease [1, 2]. The unilateral ureteral obstruction (UUO) model presents renal fibrosis unrelated to hypertension or systemic immune disease [3, 4]. Kidney obstruction after UUO results in tubular injury and cell death by apoptosis or necrosis [5–7]. One prior study has revealed the pivotal role of CD4^+^ T cells in renal fibrosis following ureteric obstruction [8]. The subtype of CD4^+^ T cell involved remains unclear. The context of T cell activation determines whether fibrocyte development is supported or blocked. Murine fibrocytes develop from a subpopulation of CD11b^+^CD115^+^Gr1^+^ monocytes under the control of CD4^+^ T cells [9]. In one mouse bronchiolitis obliterans model, the interleukin (IL)-17 level has been shown to increase locally in the absence of a change in the number of peripheral blood T helper (Th) 17 cells, whereas the number of peripheral T regulatory cells (Tregs) was shown to decrease [10]. Th17 cells, which are regulated by IL-6 stimulation, may therefore play a role in post-transplantation allograft rejection. One UUO study has identified intra-renal dendritic cells as an early source of proinflammatory mediators after acute urinary obstruction and has found that they play specific roles in the recruitment and activation of effector-memory T cells, including Th17 cells [11]. Heda Kvakan et al. have found that the immunosuppressive effects of transferred Tregs ameliorate cardiac fibrosis and improve electrical remodeling independent of their blood pressure-lowering effects [12]. Thus, Th17 cells and Tregs may be the key CD4^+^ T cells involved in organ fibrosis.

Previous studies have demonstrated the presence of links between Th17 and Treg cells. Hans J. P. M. Koenen et al. have shown that human CD25^high^FOXP3^+^ Tregs differentiate into IL-17-producing cells in the presence of rhIL-2/rhIL-15 when stimulated by antigen presenting cells [13]. They also found that the histone deacetylase (HDAC) inhibitor, trichostatin A (TSA), has a profound negative effect on the differentiation of IL-17-producing cells from Tregs and that FOXP3^+^ Treg cells. H. Jorn Bovenschen et al. also found FOXP3^+^ Regulatory T Cells in psoriasis patients readily differentiate into IL-17A-producing cells [14]. These studies have indicated that a regulatory arm of the immune response may develop along similar lines as the effector arm. These findings imply that Treg development exhibits a degree of plasticity to meet local conditions. Cells expressing IL-17 and retinoic acid receptor-related orphan receptor (RORγt) are present in lung tissue and digestive mucosal compartments [15]. Koenen HJ et al. have noted that CD4^+^FOXP3^+^ Tregs aggravate inflammation-associated Th17 cells [13]. It must be established whether FOXP3^+^IL-17^+^ T cells actually have tissue fibrosis properties. Meanwhile, although the role of HDAC inhibitors in renal fibrosis has been proposed, the involvement of FOXP3^+^IL-17^+^ T cells in the improvement of renal fibrosis mediated by HDAC inhibitors had not been described. Thus, we evaluated the role of HDAC inhibitors and FOXP3^+^IL-17^+^ T cells in renal tissues from UUO mice and in the propagation of renal fibrosis.

## Methods

### Ethic statement

This animal study was performed in strict accordance with the recommendations of the Guide for the Care and Use of Laboratory Animals of the National Laboratory Animal Center. The experimental protocol was approved by the Animal Care Committee of the Taichung Veterans General Hospital (Permit Numbers: La-101,944 and La-1,021,085). The mice had free access to tap water and standard mouse feed.

### UUO mouse model

These studies were conducted on male C57BL/6 mice (approximately 20 g in weight) obtained from the National Laboratory Animal Center (Taipei, Taiwan). The UUO mice underwent surgery as described previously [3, 16, 17]. The mice were anesthetized with Rampun (xylazine hydrochloride) and Zoletil (a mixture of tiletamine hypochloride and zolazepam hypochloride), and the left kidney and ureter were exposed via flank incision. To prevent a retrograde urinary tract infection, the left ureter was ligated with 4-0 silk at two points at the ureterovesical junction and was cut between the ligatures. Finally, the wound was closed in layers. The mice were maintained in a temperature-controlled room, and their vital signs were monitored regularly after surgery. Following UUO, TSA (0.5 mg/kg) was first administered intraperitoneally (I.P.) and then by daily injection for 7, 14 or 21 days. The mice were randomized into groups of six as follows:

1. Sham operation with vehicle (saline)
2. UUO (7, 14 or 21 days) with vehicle
3. UUO (7, 14 or 21 days) with TSA

The sham animals underwent identical surgical procedures, with the left ureter simply manipulated. The mice were sacrificed under anesthesia with Rampun and Zoletil at 7, 14, or 21 days after surgery, and the kidneys were perfused with 0.9% normal saline. Both the obstructed and contralateral kidneys were harvested.

### Reagents

We obtained anti-mouse FOXP3 fluorescein isothiocyanate (FITC) and IL-17 phycoerythrin (PE) and CD4 PE-CyChrome5 (PC5) mAbs and isotype-matched control mAbs from BD Biosciences (San Jose, CA, USA). We further verified FOXP3 mRNA expression in sorted unstimulated CD4^+^IL-17^+^ cells and obtained the same results. We used a single stain for FOXP3^+^ cells gated by CD4^+^IL-17^+^ cells.

TSA was purchased from Sigma-Aldrich (Zwijndrecht, Netherlands). Anti-phospho-signal transducer and activator of transcription 3 (STAT3) and anti-β-actin were also obtained for Western blot analysis (Abcam; Cambridge, UK).

### Histopathological and immunohistochemical (IHC) analyses

Formalin fixation and paraffin embedding of the bilateral renal specimens were performed according to previously described procedures [16]. The primary antibodies used were anti-mouse mAbs (anti-CD4 Ab, Leica, Leica Biosystems, Newcastle, UK and anti-IL-17 Ab, Abcam, Cambridge, UK), and the secondary antibody used was a rabbit monoclonal anti-FOXP3 antibody (Abcam, Cambridge, UK). Renal fibrosis was assessed with Masson trichrome staining using a commercial kit (Abcam; ab150686).

The semiquantitative evaluations and grading of the IHC staining were performed by two independent investigators. The number of CD4^+^FOXP3^+^, CD4^+^IL-17^+^, or FOXP3^+^IL-17^+^ cells was determined according to the number of positive cells per high-powered field (HPF) at 400X magnification from 10 randomly chosen fields within the same section of kidney tissue from an individual biopsy specimen [14]. The interstitial volume, α-smooth muscle actin (α-SMA) score were calculated by the point counting method in accordance with previous reports [16, 18].

### Splenic cell separation and culture

Mouse spleens and kidneys were cut into pieces, milled with a tissue grinder, and filtered using a 70-μm strainer (BD FALCON, USA). Splenocytes were isolated by Ficoll-Paque gradient centrifugation (GE Healthcare, Sweden). Mononuclear spleen cells (1 × 10^6^ cells/well) were cultured with IL-2 (10 pg/mL) (BD PHARMINGEN) in Roswell Park Memorial Institute (RPMI)-1640 medium containing 10% fetal bovine serum for 6 days and analyzed by flow cytometry.

Splenic cells were incubated with magnetic beads coated with an antibody against CD4 using a commercial magnetic-activated cell sorting (MACS) kit (Mitenyi Biotec). FOXP3^+^IL-17^+^ cells were subsequently sorted by fluorescence-activated cell sorting (FACS) (purity >95%) and checked by flow cytometry (FC500, Beckman Coulter, Fullerton, CA).

### Flow cytometry

Cells were stimulated with IL-2 for 6 days and then treated with phorbol myristate acetate (PMA) (50 ng/mL) and ionomycin (1 μg/mL) for 4 h before analysis. Splenic or renal mononuclear cells were fixed using a Cytofix/Cytoperm kit (BD Biosciences, San Jose, CA, USA) and were then stained with fluorescein-conjugated mAbs with isotopes against CD4, TGF-β1, Annexin V, FOXP3 and IL-17 (BD Biosciences). CD4^+^FOXP3^+^, CD4^+^IL-17^+^, FOXP3^+^IL-17^+^ and FOXP3^+^IL-17^+^TGF-β1^+^ T cells were identified by flow cytometry (FC500, Beckman Coulter, Fullerton, CA). The isolation of CD4^+^FOXP3^+^IL-17^+^ cells was performed with a commercial MACS kit according to the manufacturer’s instructions [19].

### Real-time quantitative reverse transcription polymerase chain reaction (qRT-PCR)

Real-time quantitative RT-PCR analyses of IL-17, TGF-β1, FOXP3 and RORγt mRNA were performed using a SYBR Green PCR Kit (Applied Biosystems, California). The specific primers used were as follows: IL-17: 5’-ATCAGGACGCGCAAACATG-3’ (sense) and 5’-TGATCGCTGCTGCCTTCAC-3’ (antisense); TGF-β1: 5’-TGCGCTTGCAGAGATTAAAA-3’ (sense) and 5’-CGTCAAAAGACAGCCACTCA-3’ (antisense); FOXP3: 5’-GGTTAGGAGACATCCATCAGG-3’ (sense) and 5’-CAGGGAGGAGTTCAGTAGAGG -3’ (antisense); RORγt: 5’-TCTGCAAGACTCATCGACAAGG-3’ (sense) and 5’-TCAGGGGATTCAACATCAGTGC -3’ (antisense); and glyceraldehyde 3-phosphate dehydrogenase (GADPH): 5’-ACTCCACTCACGGCAAATTC-3’ (sense) and 5’-TCTCCATGGTGGTGAAGACA-3’ (antisense).

The mRNA expression was calculated as the fold change using the 2^△Ct^ formula as follows: △C_T_ = △C_T_ (control)-△C_T_ (target), where C_T_ indicates the cycle threshold [19].

### Knockdown by siRNA for IL-17

One pair of small-interfering RNAs (siRNA) targeting IL-17 were synthesized by Ambion (Life Technologies Corporation, Taiwan). FOXP3^+^IL-17^+^ T cells transfected with IL-17 siRNA (20 μM) in six-well plates using Lipofectamine RNAiMAX reagent, according to the manufacturer’s protocol (Life Technologies). The Ambion’s silencer negative control siRNA was used to knockdown expression of IL-17 and IL-17, TGF-β1 protein levels were confirmed by immunoblotting, the targeted fragment of siRNA against IL-17 was: sense (5 ’→ 3’) AAGAGAUCCUGGUCCUGAAtt, anti-sense: UUCAGGACCAGGAUCUCUUgc. Quantitative data were obtained by computing densitometer and Image Quant software [19].

### Western blot analysis

Total protein was extracted from kidney tissues or isolated cells. Cell proteins were separated on a precast polyacrylamide gel (10%), transferred to a nitrocellulose membrane (Amersham Biosciences, Piscataway, NJ) and probed with primary antibodies at 4 °C overnight, followed by incubation with horseradish peroxidase (HRP)-labeled secondary antibodies for 1 h at room temperature. Protein bands were detected using enhanced chemiluminescence reagents (Amersham Biosciences, Piscataway, NJ) [19].

### Statistical analysis

The data are presented as the mean ± SD. Intergroup differences were analyzed by independent t test or analysis of variance (ANOVA) followed by Duncan’s test. A *p* value of <0.05 was considered significant.

## Results

### UUO induces juxtaglomerular (JG) hyperplasia, angiotensin II type 1 receptor (AT1R) expression and lymphocyte infiltration

**Fig. 1:**
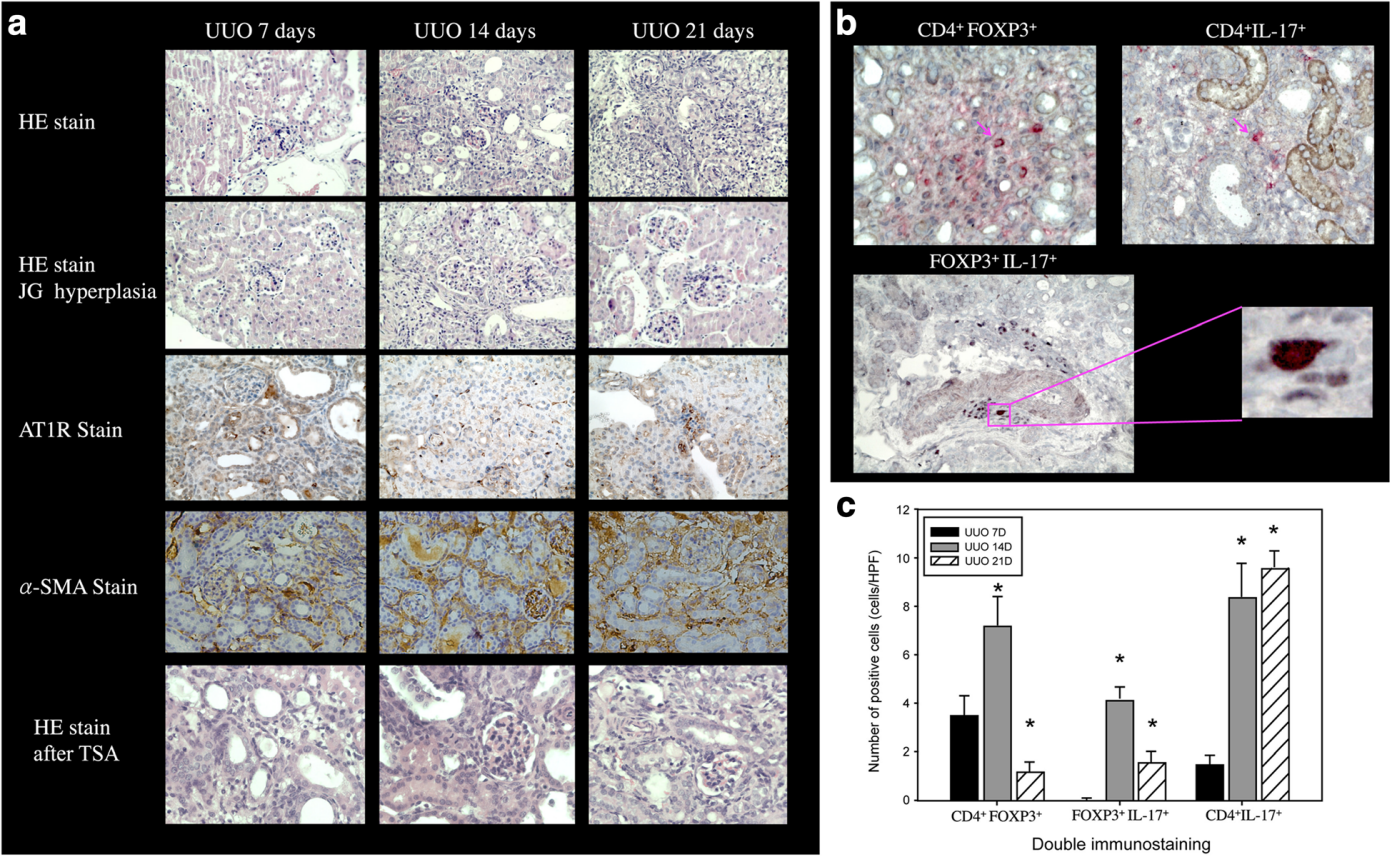
UUO-induced JG hyperplasia and AT1R and α-SMA expression. **a** Lymphocyte infiltration subsequently increased gradually, and renal fibrosis developed. TSA treatment suppressed JG hyperplasia in the UUO mice (400X). **b** CD4^+^IL-17^+^, CD4^+^FOXP3^+^ and FOXP3^+^IL-17^+^ T cells appeared in obstructed kidneys after UUO. (FOXP3^+^IL-17^+^ double stain: IL-17, blue; FOXP3, red. CD4^+^IL-17^+^ or CD4^+^FOXP3^+^ stain: CD4, red; IL-17 and FOXP3, brown, 400X). **c** The numbers of CD4^+^IL-17^+^, CD4^+^FOXP3^+^ and FOXP3^+^IL-17^+^ T cells (cells/HPF) in obstructed kidneys after UUO. *: *p* < 0.05

The top row of Fig. 1a shows hematoxylin-eosin (HE) and α-SMA staining on days 7, 14, and 21 after UUO in the kidney tissues of the UUO mice. Tubular dilatation, tubular atrophy and a widened interstitial space with increased interstitial lymphocyte infiltration were found in the obstructed kidneys. The tubulointerstitial damage progressed after UUO. We examined interstitial myofibroblasts, which are characterized by α-SMA expression (Fig. 1a). The expression of α-SMA in the cortical interstitium of the UUO mice was the highest after 21 days of UUO. Renin-angiotensin system (RAS) activation, with T-cell activation and infiltration, is thought to play a key role in the pathogenesis of renal fibrosis [20–22], but the detailed phenotypes of T-cell subsets are poorly understood. We analyzed serial changes in JG cells and AT1R expression in the kidney tissues of the UUO mice. We found progressively increasing JG cell hyperplasia in the JG apparatus, accompanied by enhanced AT1R expression in epithelial cells and lymphocytes after UUO (lines 2-3 of Fig. 1a). Progressively increasing lymphocyte infiltration was noted in the interstitium of the renal tissues after UUO. The most prominent AT1R expression in renal lymphocytes was observed at 14 days after UUO.

### The numbers of CD4^+^FOXP3^+^, CD4^+^IL-17^+^ and FOXP3^+^IL-17^+^ T cells are increased in renal tissue after UUO

A previous study has reported a pivotal role of CD4^+^ T cells in renal fibrosis; in addition, we observed lymphocyte infiltration in kidney tissues after UUO. Thus, we further analyzed the populations of CD4^+^FOXP3^+^, CD4^+^IL-17^+^ and FOXP3^+^IL-17^+^ T cell subsets in the renal tissues after UUO by double staining. CD4^+^FOXP3^+^, CD4^+^IL-17^+^ and FOXP3^+^IL-17^+^ T cell subsets were detected in the renal tissues at 14 days after UUO (Fig. 1b). The number of CD4^+^FOXP3^+^ T cells was 3.5 ± 0.8 cells/HPF in the 7-days group, and it increased to 7.2 ± 1.2 cells/HPF in the 14-days group (*p* < 0.05) but dropped to 1.2 ± 0.4 cells/HPF in the 21-days group (*p* < 0.05). The number of FOXP3^+^ IL-17^+^ T cells was 0 cells/HPF in the 7-days group, and it increased to 4.2 ± 0.5 cells/HPF in the 14-days group but fell to 1.6 ± 0.4 cells/HPF in the 21-days group (*p* < 0.05). The number of CD4^+^IL-17^+^ T cells was 1.5 ± 0.4 cells/HPF in the 7-days group, and it rose to 8.4 ± 1.4 cells/HPF in the 14-days group and to 9.6 ± 0.7 cells/HPF in the 21-days group (all *p* < 0.05, Fig. 1c). These findings show that CD4^+^FOXP3^+^, CD4^+^IL-17^+^, and FOXP3^+^IL-17^+^ T cells are vital to the pathogenesis of renal fibrosis. The number of CD4^+^IL-17^+^ cells progressively increased in ligated renal tissue. The number of CD4^+^FOXP3^+^ cells increased steadily, accompanied by the presence of FOXP3^+^IL-17^+^ cells, which appeared after 14 days but progressively decreased in number by the 21st day and were associated with renal fibrosis in the UUO mice.

### The TGF-β1 and IL-17 mRNA levels increased with duration of UUO

We next examined the expression of TGF-β1 and IL-17 mRNA in obstructed kidneys induced by UUO. TGF-β1 mRNA expression increased significantly in the UUO kidneys compared with sham-operated kidneys. After UUO, the relative level of IL-17 mRNA rose significantly in the UUO mice at 21 versus 14 and 7 days. Specifically, the relative level of TGF-β1 mRNA was the highest in the 14-days group, and it was decreased in the 21-days group. We found that the TGF-β1 mRNA and IL-17 mRNA levels were positively correlated (r^2^=0.792).

### The Th17 phenotype is generated from Treg cells, and TSA treatment reduces renal fibrosis

**Fig. 2:**
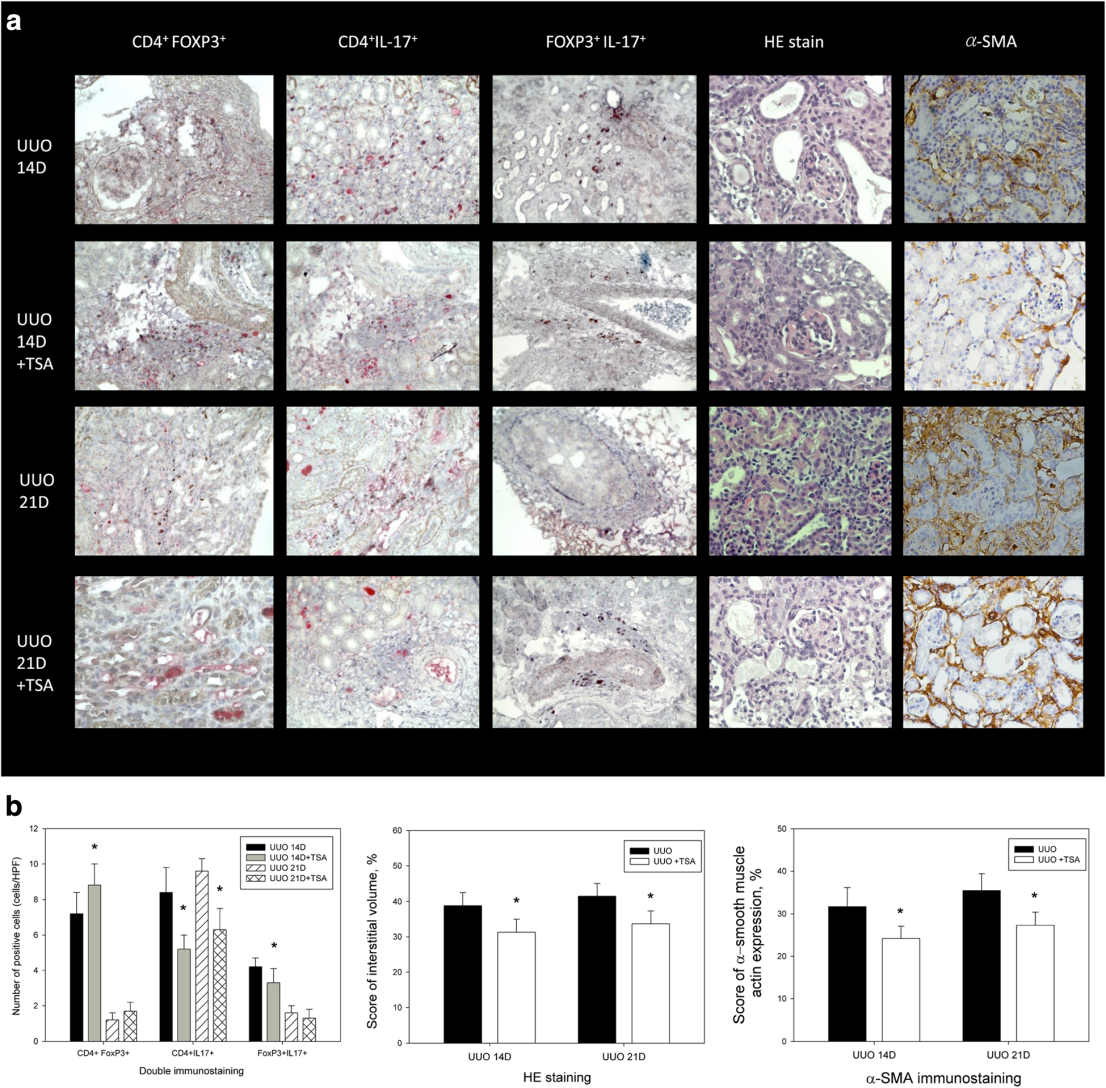
Decreased renal fibrosis after the TSA treatment in UUO. **a** HE staining and expression of FOXP3^+^IL-17^+^, CD4^+^FOXP3^+^, and CD4^+^IL-17^+^ T cells before and after TSA treatment in UUO groups after 14 and 21 days (400X). **b** The numbers of CD4^+^IL-17^+^, CD4^+^FOXP3^+^ and FOXP3^+^IL-17^+^ T cells (cells/HPF), interstitial volume score and α-SMA score in obstructed kidneys after UUO. *: *p* < 0.05

HE staining of kidney tissues from the UUO mice showed decreased fibrosis after the I.P. TSA treatment (Fig. 2a). The interstitial volume score (UUO 14D: TSA vs. UUO: 31.3 ± 3.7 vs. 38.8 ± 3.7%, *p* < 0.05, *n* = 6; UUO 21D: TSA vs. UUO: 33.7 ± 3.6 vs. 41.5 ± 3.5%, *n* = 6, Fig. 2b) and α-SMA score (UUO 14D: TSA vs. UUO: 24.2 ± 2.9 vs. 31.7 ± 4.5%, *p* < 0.05, *n* = 6; UUO 21D: TSA vs. UUO: 27.3 ± 3.1 vs. 35.5 ± 3.9%, *n* = 6, Fig. 2b) were decreased after the TSA treatment.

Double staining of FOXP3^+^IL-17^+^, CD4^+^FOXP3^+^, and CD4^+^IL-17^+^ T cells before and after the TSA treatment for 14 or 21 days in the UUO groups revealed increased numbers of CD4^+^FOXP3^+^ (UUO D14: TSA vs. UUO: 8.8 ± 1.2 vs. 7.2 ± 1.2, *p* < 0.05, *n* = 6; UUO D21: TSA vs. UUO: 1.7 ± 0.5 vs. 1.2 ± 0.4, *n* = 6, Fig. 2b) and decreased numbers of CD4^+^IL-17^+^ (UUO D14: TSA vs. UUO: 5.2 ± 0.8 vs. 8.4 ± 1.4, *p* < 0.05, *n* = 6; UUO D21: TSA vs. UUO: 6.3 ± 1.2 vs. 9.6 ± 0.7, *p* < 0.05, *n* = 6, Fig. 2b) and FOXP3^+^IL-17^+^ (UUO D14: TSA vs. UUO: 3.3 ± 0.8 vs. 4.2 ± 0.5, *p* < 0.05, *n* = 6; UUO D21: TSA vs. UUO: 1.3 ± 0.5 vs. 1.6 ± 0.4, *n* = 6, Fig. 2b) T cells. CD4^+^FOXP3^+^IL-17^+^ T cells in single-cell suspensions were generated from renal tissues after UUO from mice with and without the TSA treatment.

**Fig. 3:**
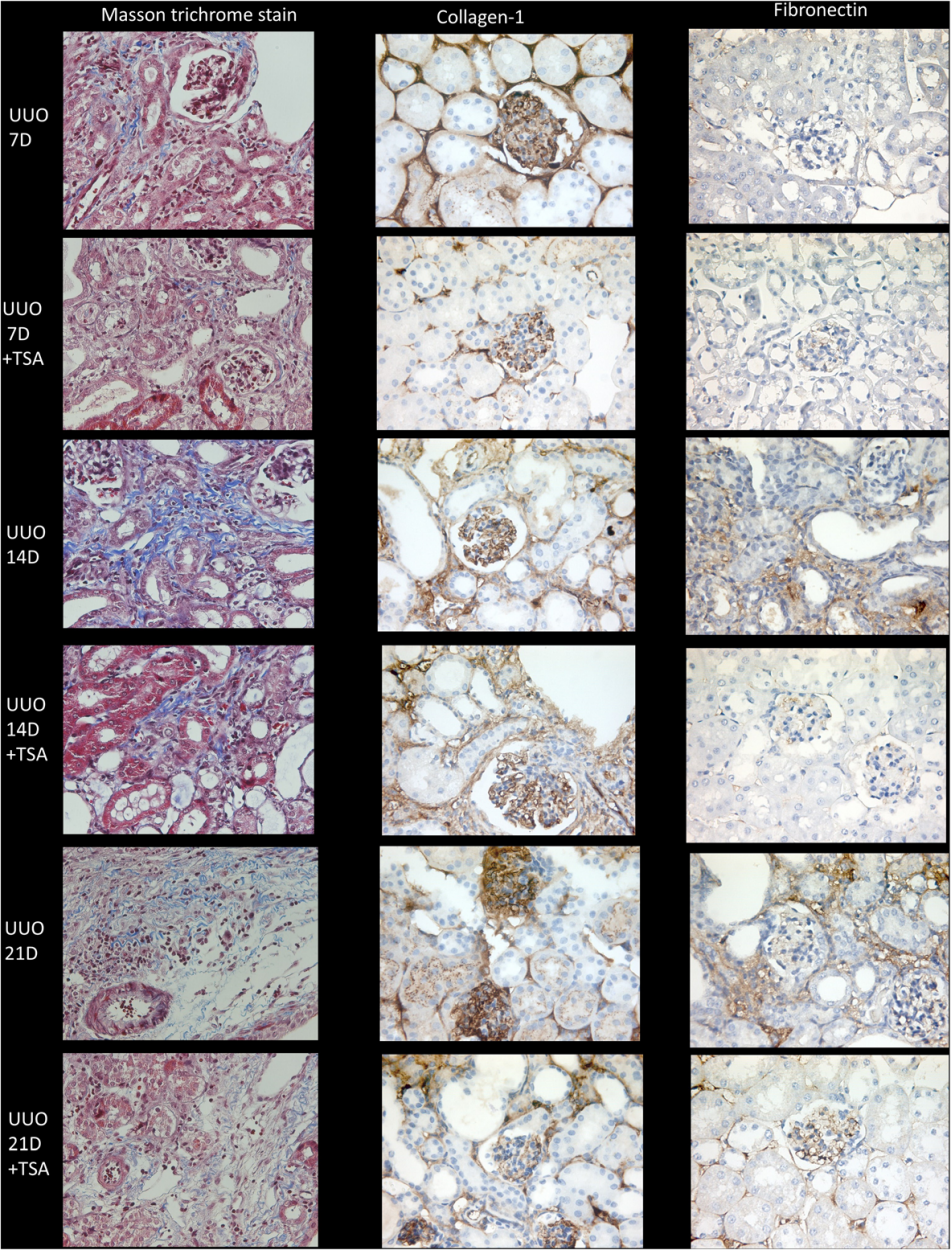
Masson trichome staining revealed increased collagen deposition in the renal tissues after UUO for 7, 14 and 21 days and decreased after TSA treatment (400X). IHC for type 1 collagen and fibronectin were also revealed positive stains after UUO for 7, 14 and 21 days (400X)

Masson trichrome staining revealed increased collagen deposition in the renal tissues after UUO for 7, 14 and 21 days (Fig. 3). The collagen deposition decreased after TSA treatment. IHC for type 1 collagen and fibronectin were also showed positive stain after UUO for 7, 14 and 21 days (Fig. 3).

**Fig. 4:**
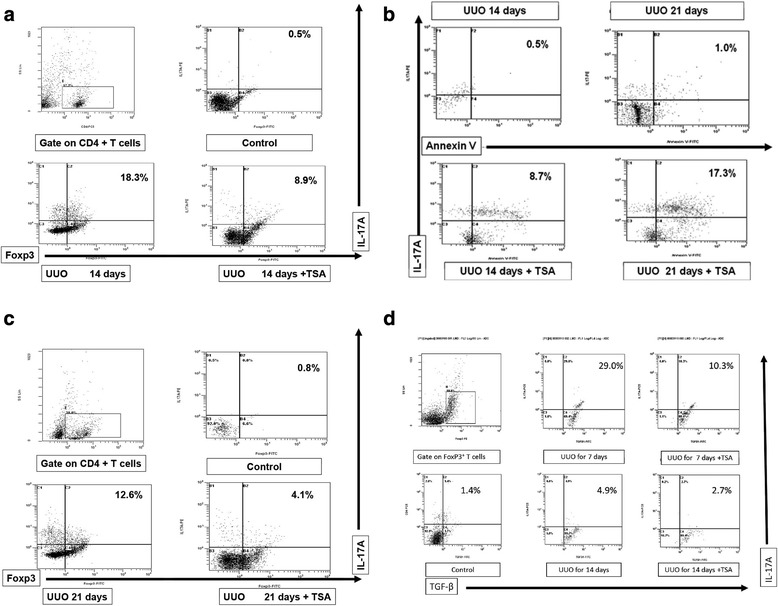
CD4^+^FOXP3^+^IL-17^+^ T cells in a single-cell suspension from renal tissues from UUO mice treated with or without TSA. The cells were subjected to membrane and intracellular staining and were analyzed by fluorescence-activated cell sorting (FACS). **a**-**b** Fresh single-cell suspensions prepared from 14- and 21-days UUO ligated kidneys (CD4 gated). **c** TSA increased the appearance of apoptotic IL-17^+^Annexin^+^ cells (CD4 gated). **d** Flow cytometry (CD4 gated) detected splenic IL-17^+^FOXP3^+^TGF-β1^+^ T cells, the percentage of which was increased after UUO for 7 or 14 days. After TSA treatment, this percentage decreased

Flow cytometry detected CD4^+^FOXP3^+^IL-17^+^ T cells after 14 (Fig. 4a, bottom left) and 21 days (Fig. 4b, bottom left). Following treatment with TSA, the number of CD4^+^FOXP3^+^IL-17^+^ T cells declined both after 14 days (without TSA vs. TSA: 18.8 ± 2.1 vs. 8.2 ± 1.4%, *p* < 0.05, *n* = 6, Fig. 4a, bottom right) and after 21 days (without TSA vs. TSA: 12.4 ± 2.3 vs. 4.4 ± 0.6%, *p* < 0.05, *n* = 6, Fig. 4b, bottom right). TSA enhanced the appearance of apoptotic IL-17^+^Annexin^+^ cells (day 14: without TSA vs. TSA: 0.5 ± 0.1 vs. 8.4 ± 1.2%, *p* < 0.05, *n* = 6; day 21: without TSA vs. TSA: 0.9 ± 0.3 vs. 17.6 ± 2.1%, *p* < 0.01, *n* = 6; Fig. 4c).

### Up-regulation of FOXP3 and down-regulation of RORγt mRNA expression after UUO and was modifiable by TSA treatment

**Fig. 5:**
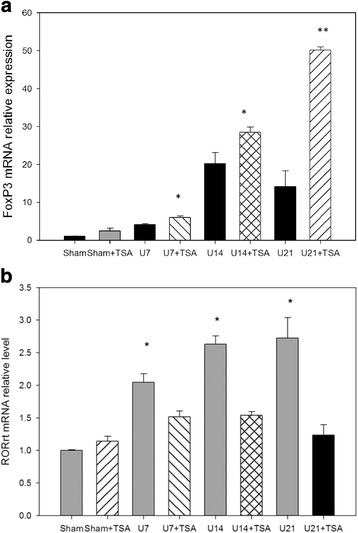
UUO increased FOXP3 (**a**) and RORγt (**b**) mRNA expression levels. After TSA treatment, the FOXP3 mRNA expression levels increased further. *: *p* <0.05; **: *p* < 0.001

**Fig. 6:**
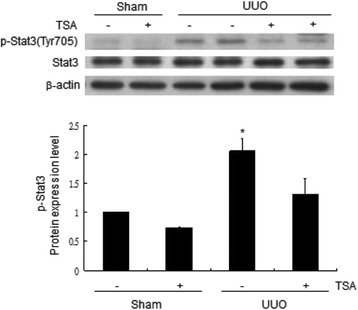
TSA inhibited STAT3 phosphorylation in a UUO mouse model by western blotting 14 days after UUO. *: *p* < 0.05

**Fig. 7:**
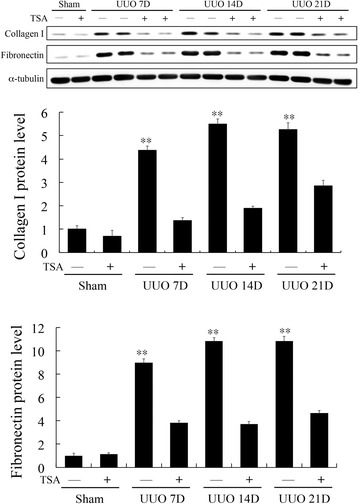
Western blotting of type 1 collagen and fibronectin. Type 1 collagen and fibronectin are markers for renal fibrosis. The result showed that TSA inhibited the increased protein level of fibronectin and type 1 collagen induced by UUO. **: *p* < 0.001

Figure 5 shows increased FOXP3 and RORγt mRNA expression in kidney tissue after UUO. After the TSA treatment, the FOXP3 mRNA expression level increased (Fig. 5a). RORγt mRNA expression was continuously suppressed by TSA (Fig. 5b). STAT3 activation involved in renal fibrosis had been reported [23]. Our result of western blot showed TSA inhibited STAT3 phosphorylation after UUO for 14 days (*p* < 0.05, Fig. 6). Western blot was also arranged to analyze the protein levels of fibronectin and type 1 collagen, which are markers for renal fibrosis. It also showed TSA inhibited the increase of fibronectin and type 1 collagen induced by UUO (*p* < 0.001, Fig. 7).

### Progressive renal fibrosis was associated with the loss of CD4^+^FOXP3^+^IL-17^+^ T cells in single-cell suspensions of splenic cells

**Fig. 8:**
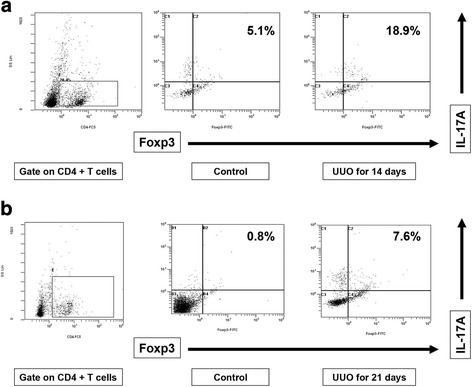
Flow cytometry data (CD4 gated), showing that the number of CD4^+^FOXP3^+^IL-17^+^ cells among splenic cells was increased after 14 days (**a**) but was decreased after 21 days (**b**)

We further evaluated CD4^+^FOXP3^+^IL-17^+^ T cells by flow cytometry in single-cell suspensions of splenic cells, which have been used previously [19]. We examined the serial changes in CD4^+^FOXP3^+^IL-17^+^ T cells in single-cell suspensions prepared from splenic cells of the UUO mice. Flow cytometry revealed the presence of CD4^+^IL-17^+^FOXP3^+^ T cells after 14 days (UUO vs. controls: 17.2 ± 2.4 vs. 4.8 ± 2.1%, *p* < 0.01, *n* = 6, Fig. 8a), but they decreased in number after 21 days (UUO vs. controls: 7.8 ± 0.8 vs. 0.9 ± 0.3%, *p* < 0.05, *n* = 6, Fig. 8b). These findings corresponded with the double immunostaining findings in the renal tissues.

### Splenic FOXP3^+^IL-17^+^ T cells express TGF-β1 and knockdown by siIL-17

**Fig. 9:**
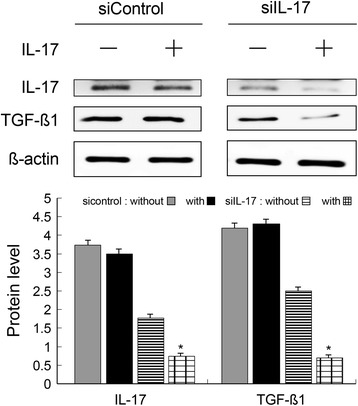
Splenic FOXP3^+^IL-17^+^ T cells express TGF-β1 and knockdown by siIL-17. Western blot showed IL-17 expression significantly decreased accompany with decreasing TGF-β1. These results demonstrated that IL-17 regulates TGF-β1 expression in FOXP3^+^IL-17^+^ T cells. *: *p* < 0.05

Flow cytometry detected the presence of splenic IL-17^+^FOXP3^+^TGF-β1^+^ T cells after 7 days (UUO vs. controls: 26.4 ± 15.0 vs.1.4 ± 0.5%, *p* < 0.01, *n* = 6), but they decreased in number after 14 days (UUO vs. controls: 4.9 ± 1.9 vs. 1.4 ± 0.5%, *n* = 6). TSA reduced the number of IL-17^+^FOXP3^+^TGF-β1^+^ T cells after 7 days (TSA vs. UUO: 10.3 ± 4.2 vs. 26.4 ± 15.0%, *p* < 0.01, *n* = 6) and after 14 days (TSA vs. UUO: 2.4 ± 0.6 vs. 4.9 ± 1.9%, *p* < 0.01, *n* = 6, Fig. 4d). To investigate whether FOXP3^**+**^IL-17^**+**^ T cell is responsible for TGR-β1 expression, we assessed TGF-β1 in sorted Day 7 splenic FOXP3^**+**^IL-17^**+**^ T cells by transfecting cell with a control and a IL-17 siRNA. We found that IL-17 expression significantly decreased accompany with decreasing TGF-β1(Fig. 9). These results demonstrated that IL-17 regulates TGF-β1 expression in FOXP3^**+**^IL-17^**+**^ T cells.

### Increased TGFβ1 expression in splenic CD4^+^FOXP3^+^IL-17^+^ T cells after 7 days and loss of FOXP3 expression with enhanced expression of IL-17 after 14 days in UUO mice

**Fig. 10:**
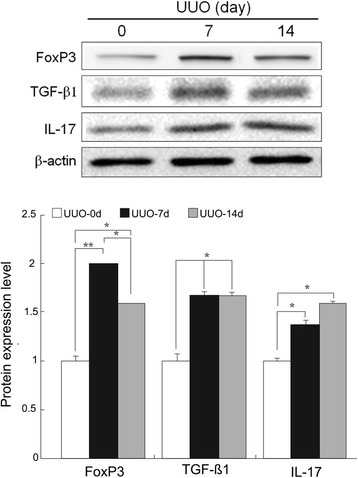
Western blotting of FOXP3^+^IL-17^+^ T cells also showed increased TFG-β1 expression after UUO. The gradual conversion of FOXP3^+^ Treg cells into IL-17-producing cells with a loss of FOXP3 expression and enhanced IL-17 expression after 14 days was also noted. *: *p* < 0.05; **: *p* < 0.001

Splenic cells were incubated with magnetic beads coated with an antibody against CD4. FOXP3^+^IL-17^+^ cells were isolated and sorted (purity >95%) and were then checked by flow cytometry. Western blotting for TGFβ1, FOXP3, and IL-17 expression in splenic CD4^+^FOXP3^+^IL-17^+^ T cells on days 0, 7, and 14 showed that the increased TGFβ1 expression occurred after 7 and 14 days (Fig. 10), as determined by flow cytometry (*p* < 0.05). We observed the gradual conversion of FOXP3^+^ Treg cells into IL-17-producing cells with a loss of FOXP3 expression and enhanced IL-17 expression after 14 days. These findings imply that the transformation of CD4^+^FOXP3^+^IL-17^+^ T cells into CD4^+^IL-17^+^ T cells may be linked to the TGF-β1 secretion that contributes to the progression of renal fibrosis after UUO and can be suppressed by TSA.

## Discussion

We observed lymphocyte infiltration in obstructed kidneys after UUO. The infiltrating lymphocytes included CD4^+^IL-17^+^, CD4^+^FOXP3^+^, and IL-17^+^FOXP3^+^ T cells in the obstructed kidneys after UUO, as demonstrated by histopathological and IHC analyses, indicating that CD4^+^ T cell differentiation occurs after UUO. We also detected splenic CD4^+^IL-17^+^FOXP3^+^ T cells after 14 days, but they decreased in number after 21 days. These findings provide evidence that CD4^+^IL-17^+^FOXP3^+^ T cells appear in both the spleen and kidneys. Involvement of splenocytes suggests the systemic involvement of CD4^+^IL-17^+^FOXP3^+^ T cells after UUO, but this requires further examination in the future to obtain stronger evidence. We measured TGF-β1 mRNA expression and found that it was positively correlated with IL-17 mRNA expression in UUO renal tissues, suggesting a link between Th17 cells and renal fibrosis. We perfused renal arteries with 0.9% normal saline and washed out circulating blood before harvesting to prevent contamination of the renal tissues with systemic mononuclear cells. With minimal contamination, the accuracy of flow cytometry for the kidney cells was high, and the CD4^+^FOXP3^+^, CD4^+^FOXP3^+^IL-17^+^ and CD4^+^IL-17^+^ T cells were unlikely to have resulted from systemic blood contamination.

Prominent roles of Treg and Th17 cells in chronic diseases have been reported in previous studies. Nakagiri et al. have shown that IL-17 production increases locally in the absence of a change in the number of peripheral blood Th17 cells and that the number of peripheral Tregs decreases after allografts in a mouse bronchiolitis obliterans model. Adoptive Treg cell transfer has been shown to be beneficial in animal models of diabetes, arthritis, transplantation and renal disease [24–28]. Heda Kvakan et al. have demonstrated that Treg cell transfer reduces cardiac hypertrophy and fibrosis [12]. Prior studies have shown that human Tregs can be converted into Th17 cells [15, 29, 30]. IL-17 has been identified as being fundamental to TGF-β1-derived fibrosis [31]. In our study, we detected IL-17^+^FOXP3^+^ T cells after stimulation of CD4^+^FOXP3^+^ Tregs in a UUO mouse model. We demonstrated that the number of IL-17^+^FOXP3^+^ cells, as well as the severity of renal fibrosis, were decreased with TSA treatment. Masson trichrome staining also revealed collagen deposition in renal tissues decreased after TSA treatment. Western blot showed the protein levels of type 1 collagen and fibronectin were decreased after TSA treatment which represented the anti-fibrotic effects of TSA treatment. These results are compatible with the pervious study [23]. The anti-fibrotic effects of TSA may be related to suppression of the conversion of Tregs into Th17 cells. Expression of FOXP3 and RORγt mRNA was increased after UUO in this study. After TSA treatment, FOXP3 mRNA expression was elevated. RORγt mRNA expression rose after UUO but fell after TSA treatment. FOXP3 and RORγt are the transcription factors present in CD4^+^FOXP3^+^ and CD4^+^IL-17^+^ T cells, respectively. The anti-fibrotic effects of TSA may arise from the up-regulation of CD4^+^FOXP3^+^ T cells and the down-regulation of CD4^+^IL-17^+^ T cells. We observed that TSA increased the number of IL-17^+^Annexin^+^ apoptotic cells. The finding of the inhibition of STAT3 phosphorylation supported our hypothesis that a mechanism underlying the anti-fibrotic effect of TSA induces CD4^+^IL-17^+^ T cell apoptosis. TSA has been shown to prevent the differentiation of Tregs into Th17 cells in psoriasis patients in a previous study [14]. In addition, an earlier study has revealed that TSA inhibition induces anti-fibrotic activity by inactivating renal interstitial fibroblasts and inhibiting renal tubular cell death. STAT3 may mediate the actions of HDACs [23]. Mao-yin Pang has suggested that TSA has a pharmacological anti-fibrotic effect that occurs via the suppression of renal interstitial fibroblasts and the inhibition of renal tubular cell death [23]. Yoshikawa et al. have examined the effect of TSA on the epithelial-mesenchymal transition (EMT) in cultured tubular epithelial cells and have found that TSA prevents the TGF-β1-induced EMT [32]. They have revealed that TSA induces the expression of inhibitor of DNA binding 2 (ID2) and bone morphogenetic protein-7 (BMP-7), which are both inhibitors of TGF-β1 signaling. Imai N. et al. have shown that TSA treatment attenuates the progression of proteinuria and glomerulosclerosis in a mouse model of nephrotoxic serum nephritis (NTN) by promoting BMP-7 expression [33]. Hence, HDACs may contribute to renal fibrosis through multiple mechanisms.

To date, data on the correlation between the plasticity of Treg development and renal fibrosis are limited. We have demonstrated a CD4^+^FOXP3^+^ Treg effector differentiation program that yields Th17^+^ cells that induce TGF-β1 mRNA expression and renal fibrosis in UUO kidneys. Our data suggest that epigenetic modifications underlie this phenomenon. Flexibility in Treg differentiation programming may have evolved to anticipate local microenvironmental regulation. Notably, the presence of TGF-β1-secreting FOXP3^+^IL-17^+^ T cells in the proinflammatory environment suggests a role for these cells in the conversion of Tregs into IL-17-producing cells, as well as TGF-β1-producing cells, inadvertently contributing to the perpetuation of both inflammatory conditions and remodeling. These observations provide insights into a previously unreported mechanism of inflammation and remodeling. However, further research is necessary to evaluate the interaction between IL-7 and TGF-β1 and its signaling pathways.

## Conclusions

In summary, UUO induces lymphocyte invasion. Tregs differentiate into Th17 cells in renal tissue after UUO. The numbers of both TGF-β1-producing FOXP3^+^IL-17^+^ and Th17 cells are positively correlated with TGF-β1 mRNA expression and fibrosis in UUO kidneys. FOXP3^**+**^IL-17^**+**^ T cell expressed TGF-β1 both in vitro and in vivo, and TGF-β1 expression was significantly knocked down by IL-17 siRNA in vitro. Treg plasticity is involved in the balance between FOXP3 and RORγt signaling, as well as remodeling. Inhibition of HDAC activity by TSA results in the prevention of fibrosis and the conversion of Tregs into IL-17^+^ Tregs. In short, therapeutic benefits may be attained via epigenetic modifications.

## Abbreviations

AT1R: Angiotensin II type 1 receptor
EMT: Epithelial-to-mesenchymal transformation
FOXP3: Forkhead box P3 (FOXP3)
HDAC: The histone deacetylase inhibitor
HPF: High-powered field
JG: Juxtaglomerular
qRT-PCR: Real-time quantitative reverse transcription polymerase chain reaction
RORγt: Retinoic acid receptor-related orphan receptor
STAT3: Signal transducer and activator of transcription 3
Th17: T helper 17 cells
Treg: T regulatory cells
TSA: Trichostatin A
UUO: Unilateral ureteral obstruction
α-SMA: Alpha-smooth muscle actin
